# Modulation of Cell Behavior by 3D Biocompatible Hydrogel Microscaffolds with Precise Configuration

**DOI:** 10.3390/nano11092325

**Published:** 2021-09-07

**Authors:** Wei-Cai Zhang, Mei-Ling Zheng, Jie Liu, Feng Jin, Xian-Zi Dong, Min Guo, Teng Li

**Affiliations:** 1 Laboratory of Organic Nano Photonics and CAS Key Laboratory of Bio-Inspired Materials and Interfacial Science, Technical Institute of Physics and Chemistry, Chinese Academy of Sciences, No. 29, Zhongguancun East Road, Beijing 100190, China; zhangweicai17@mails.ucas.ac.cn (W.-C.Z.); liujieby@mail.ipc.ac.cn (J.L.); jinfeng@mail.ipc.ac.cn (F.J.); dongxianzi@mail.ipc.ac.cn (X.-Z.D.); hsdguom@163.com (M.G.); liteng19@mails.ucas.ac.cn (T.L.); 2 School of Future Technologies, Yanqihu Campus, University of Chinese Academy of Sciences, Beijing 101407, China

**Keywords:** 3D hydrogel microscaffold, two-photon polymerization, porosity, cell behavior, fibroblasts

## Abstract

Three-dimensional (3D) micronano structures have attracted much attention in tissue engineering since they can better simulate the microenvironment in vivo. Two-photon polymerization (TPP) technique provides a powerful tool for printing arbitrary 3D structures with high precision. Here, the desired 3D biocompatible hydrogel microscaffolds (3D microscaffold) with structure design referring to fibroblasts L929 have been fabricated by TPP technology, particularly considering the relative size of cell seed (cell suspension), spread cell, strut and strut spacing of scaffold. Modulation of the cell behavior has been studied by adjusting the porosity from 69.7% to 89.3%. The cell culture experiment results reveal that the obvious modulation of F-actin can be achieved by using the 3D microscaffold. Moreover, cells on 3D microscaffolds exhibit more lamellipodia than those on 2D substrates, and thus resulting in a more complicated 3D shape of single cell and increased cell surface. 3D distribution can be also achieved by employing the designed 3D microscaffold, which would effectively improve the efficiency of information exchange and material transfer. The proposed protocol enables us to better understand the cell behavior in vivo, which would provide high prospects for the further application in tissue engineering.

## 1. Introduction

Damage or defect of tissues and organs from traumas and tumors seriously threaten human health, thus the repair and reconstruction of these defects has emerged as one of the challenges in modern medicine. Tissue engineering is based on a small number of cells and supported by biological materials for trauma-induced repair and in vitro tissue reconstruction [1,2]. Fibroblasts are often used to enhance the osteogenic differentiation [3], and promote wound healing and capillary degeneration [4] due to their wide distribution in vivo, high number, and great proliferative and multi-directional differentiation potential. With the continuous development of three-dimensional (3D) scaffolds that mimic the real microenvironment in vivo [5,6,7,8], investigations of scaffold-induced cell behavior for wound repair and tissue healing are becoming a new research focus [9,10,11].

Previous studies have considered the induction behavior of cells modulated by the parameters of 3D scaffolds such as pore size [12,13,14,15], pore shape [16,17,18], surface roughness [19,20,21], support strength [22,23], and wettability [21]. A large number of studies have focused on the influence of the porosity and pore size of the 3D microscaffold on cell behaviors. However, it is worth mentioning that the struts of these scaffolds mostly overlap in the vertical direction, forming a space similar to a “patio” [24,25], and the struts and pores are either much smaller than the cell seed (cell suspension) [26], or much larger than the spread cells [27,28,29]. The reported small scaffolds provide a patterned surface for the cells, in which cell growth resembles that on flat substrate. In the case of scaffold with large pore size and strut, cell seeds either fall on the strut or into the patio, which is still a 2D contact for single cell. Cell adhesion, proliferation, migration and differentiation in these scaffolds are also similar to those on the 2D substrate. Cells on such scaffolds gradually lose their biological characteristics as those in vivo. Therefore, it is important to modulate the cell behavior through 3D architecture since it can well mimic the microenvironment in vivo. However, it is still a challenge to fabricate the 3D hydrogel cell microscaffold with the precise configuration comparable to the subcellular structure. Two-photon polymerization (TPP) technique is promising for fabricating arbitrary 3D microstructures with high spatial resolution due to the nonlinear optical effect and 3D fabrication capability [30,31]. High resolution at hundred-nanometer scale can be achieved, which can guarantee the precise configuration of the microscaffold [32]. TPP has been employed in fabricating functional photonic devices [33,34] and biocompatible hydrogels [35,36] by using organic materials or composites. Recently, our group has carried out many studies in the design and fabrication of hydrogels in aqueous phase, which is promising for the potential application in tissue engineering [30,32,35,37].

In this study, a series of biocompatible 3D hydrogel microscaffolds have been proposed and fabricated by TPP, considering the size relationship of cells and the architecture parameters of scaffold. Line array and scaffold with small pore size and thin strut (ST scaffold) were also fabricated for comparison. The difference of cell growth on different microstructures was first investigated. Then, the ability to modulate fibroblast morphology and adhesion by using 3D microscaffolds with porosity values from 69.7% to 89.3% was studied. Finally, 3D cell distribution and interactions between cells and scaffolds were evaluated. The results illustrate that the microfilament cytoskeleton on 3D microscaffold gradually changes from bar-shaped, to rectangular, and finally to square as the porosity increases. The 3D microscaffold not only allows for 3D growth of single cells, but also contributes to 3D distribution. A single cell in a 3D microscaffold would rely on the support of the strut and extend more lamellipodia along the strut. A static 3D distribution was also achieved, since the population of cells would adhere to any layer in this microscaffold. These are beneficial for cell communication, and thus would result in more efficient information exchange and material exchange between cells. 

## 2. Materials and Methods

### 2.1. Materials and Preparation of Hydrogel

PEGDA (Mn = 700), the 2-benzyl-2-(dimethylamino)-4′-morpholinobutyrophenone (Irgure 369), and Benzil were purchased from Sigma Aldrich Reagent Company (St. Louis, Missouri, MO, USA). PE-3A (LOT. No. 0072994) was obtained from KYOEISHA (Osako, Japan). Hyclone Dulbecco’s Modified Eagle Medium (DMEM)/High Glucose culture medium was purchased from GE Healthcare Life Sciences (Logan, Utah, USA). Penicillin-streptomycin and DAPI probe were purchased from Beijing Solarbio Science and Technology Co., Ltd. (Beijing, China), and fetal bovine serum was purchased from TransGen Biotech (Beijing, China). Chemicals such as NaCl, KCl, KH_2_PO_4_, and Na_2_HPO_4_ were purchased from Amresco Company (Solon, Ohio, USA). Ethanol was purchased from Sinopharm Chemical Reagent Co., Ltd. (Shanghai, China). Ultrapure water was produced by Millipore Milli-Q water preparation instrument (MQ water resistivity >18 MΩ·cm, Molsheim, France). ActinRed 555 was purchased from Thermo Fisher scientific corporation (Eugene, OR, USA). Fibroblast L929 was purchased from Shanghai cell bank, Chinese Academy of Sciences (Shanghai, China). All of the regents were used without purification in the experiment.

**Figure 1:**
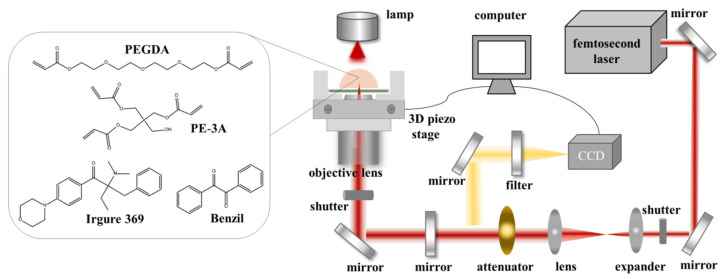
Chemical structure of the photoresist and the schematic of femtosecond laser TPP system.

In this study, PEGDA is used as monomer, and PE-3A is used as crosslinker, while Irgure 369 and Benzil in a mixing weight ratio of 1:1 are used as photoinitiators. According to the previous study in our research group [26], the scaffold shows enough Young’s modulus for cell culture when the weight ratio of PEGDA and PE-3A is 2:3. Therefore, the photoresist using PEGDA, PE-3A and the mixing photoinitiators with the weight ratios (wt. %) of 39.2: 59.2: 0.8: 0.8 were prepared. Figure 1 (left) and Table S1 display the chemical structures and the components of the monomer, crosslinker and the photoinitiators. 

### 2.2. Fabrication of Different Microstructures

All of the microstructures were fabricated by TPP using the as-prepared photoresist. The optical setup of TPP technique is shown in Figure 1 (right). The TPP optical system consists of femtosecond laser source, laser alignment part, 3D piezo stage, real-time observation system, computer and software control system. The femtosecond laser is mainly composed of an optical resonator and a pump laser light source, with a center wavelength of 780 nm, a repetition frequency of 80 MHz, and a pulse width of 120 fs. The laser power is precisely controlled by an attenuator. Photoresist was dropped directly onto a cleaned glass substrate or glass Petri dish above the stage. Femtosecond laser was tightly focused into the photoresist through an oil immersion objective lens (Olympus, Tokyo, Japan) with numerical aperture (N. A.) of 1.42 and magnification of 60×. During the TPP process, a computer-controlled *xyz* piezo stage (P-563.3 CL, Physik Instrumente, Karlsruhe, Germany) was used to move the sample in accordance with the trajectory of the preset pattern. After the fabrication, the unpolymerized photoresist was removed by ethanol and then the desired structures were retained.

### 2.3. Cell Culture

At least three 3D microscaffolds were prepared at the same size parameters in each Petri dish. Line arrays and ST scaffold were used as control, occupying the same area in the *xy* plane as 3D microscaffolds. Before cell culture, Petri dishes with microstructures were rinsed three times by using PBS buffer. Then, it was sterilized with 70% ethanol and UV light for 5 min and 30 min, respectively [38].

Fibroblast L929 cells were cultured in Hyclone DMEM/High Glucose medium that supplemented with 10% fetal bovine serum and 1% penicillin-streptomycin [39]. The culture medium was replaced every day. Fibroblast L929 cells were then seeded (constant density of 5 × 10^5^ cells/cm^2^) on the glass-bottom Petri dish (35 mm × 12 mm Style, NEST, Cat. No.: 706001) with microscaffolds and incubated at 37 °C, 5% CO_2_ in humidified incubator for 48 h [26,40]. 

### 2.4. Laser Scanning Confocal Microscope (LSCM) and Scanning Electron Microscopy (SEM) Characterization

Laser scanning confocal microscope (LSCM, Nikon, A1R MP, Tokyo, Japan) was used for observing the cell behavior, for which the excitation wavelengths are 405 nm (Coherent, CUBE, Singapore), 488 nm (Coherent, Sapphire, Germany) and 561 nm (Coherent, Sapphire, Germany), respectively. Oil immersion objective lens of 60× (Nikon) was used. After 48 h of cell culture, the medium was removed, and cells were rinsed three times with PBS buffer. Cells were fixed by using 4% paraformaldehyde and permeabilized with 0.1% Triton X-100. Then, 2 drops of ActinRed 555 per mL of media were added. The sample was incubated at room temperature and protected from light for 30 min, and then re-stained with DAPI (ready-to-use) staining solution for 10 min. Confocal fluorescence images were recorded by using LSCM after staining. The fluorescence image of F-actin was excited by 561 nm and recorded in the wavelength range of 570–620 nm, while the nuclei were excited by 405 nm and recorded in 410–460 nm.

Cells cultured on 3D microscaffolds were also observed through a field emission scanning electron microscope (SEM, Hitachi S-4800, Tokyo, Japan). Before SEM observation, the glasses and Petri dishes with 3D scaffolds and cells were coated with 10 nm of gold film. Please note that, the samples with cells need to be dehydrated in steps with ethanol (20–100%, +10% for 20 min per concentration). After the dehydration, ethanol was removed. Then, the samples were dried in air at room temperature for 10 min before gold sputtering.

### 2.5. Mechanical Performance

Moreover, Dimension FastScan Bio atomic force microscope (AFM, Bruker, Berlin, Germany) was employed to study the mechanical properties of the as-prepared hydrogel microstructures. Before the measurement, the glass substrate with cubic microstructures (more than 20 μm × 20 μm × 2 μm) was immersed in water for 5 min, until the cubic microstructures absorbed enough water molecules and reached a balanced state, and then the probe of the atomic force microscope was used to measure the Young’s modulus. The working tip radius was 6 μm. The force applied during characterizing was 0.36–9.27 nN. The spring constant was 0.1 N/m. The sample Poisson’s ratio was 0.5. The modulus fit model was Hertzian model (Spherical). Subsequently, the Young’s modulus was analyzed by NanoScope Analysis 1.9 software (Bruker, Berlin, Germany).

## 3. Results and Discussion

### 3.1. Processing Performance of Hydrogel-Based Photoresist

**Figure 2:**
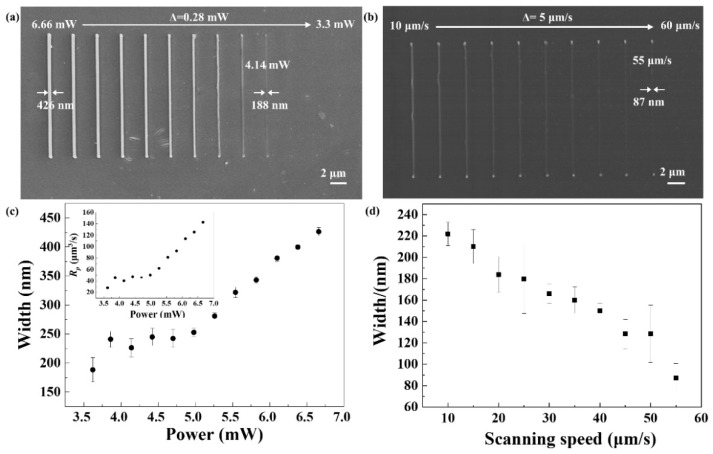
(**a**) SEM image of nanolines achieved with laser power ranging from 6.66 to 3.3 mW at 10 μm/s. (**b**) SEM image of nanolines achieved with scanning speed ranging from 10 to 60 μm/s at 6.40 mW. (**c**) The dependence of line width and polymerization rate (inset) on laser power, respectively. (**d**) The dependence of line width on scanning speed.

To evaluate the processing performance of as-prepared hydrogel-based photoresist, TPP processing threshold and the best resolution were firstly explored through fabricating nanolines. When the scanning speed was set as 10 μm/s, the laser power changed from 6.66 to 3.3 mW, with an interval of 0.28 mW. The laser power was measured before the objective lens. SEM images of the nanolines are shown in Figure 2a, which indicates the threshold of PEGDA-based photoresist is 4.14 mW. The relationship between line width and laser power was studied in Figure 2c. It illustrates that the smaller the power, the smaller the line width. With the laser power decreases from 6.66 to 4.14 mW, the line width decreases from 426 to 188 nm. Simultaneously, the polymerization rate (*R_p_*) was investigated according to the equation *R_p_* = π(*d/*2)^2^*V_s_*, where *d* is the line width of the nanolines and *V_s_* stands for the laser scanning speed [38]. The inset in Figure 2c shows the diagram of polymerization rate to laser power, which increases from 0.28 to 1.43 μm^3^/s at the constant scanning speed of 10 μm/s. Subsequently, laser power was set at a constant value of 6.40 mW, while scanning speed ranged from 10 to 60 μm/s, with an interval of 5 μm/s during the laser direct writing of nanolines. Figure 2b,d show the SEM images of nanolines and the variation of line width with scanning speed, respectively. The results indicate that it is difficult to initiate the polymerization reaction in the as-prepared photoresist in a single scanning at laser power of 6.40 mW and scanning speed of 60 μm/s. The best resolution was obtained at 55 μm/s, with a minimum line width 87 nm, which could guarantee the fabrication of high-resolution scaffolds. 

### 3.2. Mechanical Properties

**Figure 3:**
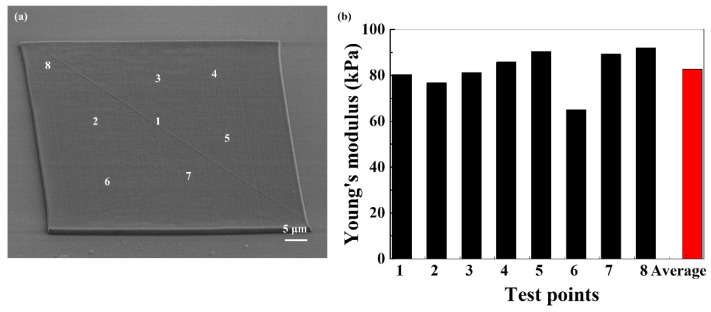
(**a**) SEM image of cubic microstructure. (**b**) The corresponding Young’s modulus of different location measured by AFM.

The Young’s modulus was studied in order to better understand the mechanical properties of the hydrogel scaffold. Several cubic microstructures with sizes of 60 μm × 60 μm × 3 μm (*l* × *w* × *h*) were fabricated, where *l* is the length of the cubic microstructure, and *w* and *h* are the width and height, respectively. Each cube was measured at at least five points to determine the Young’s modulus. Figure 3a is the SEM image of the cubic microstructure. The corresponding Young’s modulus is shown in Figure 3b, ranging from 65 to 92.1 kPa under water. The Young’s modulus measured at different locations varied very little, revealing that the entire cube remains substantially stable in water. The average Young’s modulus is 82.65 kPa, which is comparable to that of tissues [41,42].

### 3.3. Design and Fabrication of Microstructures by TPP

**Figure 4:**
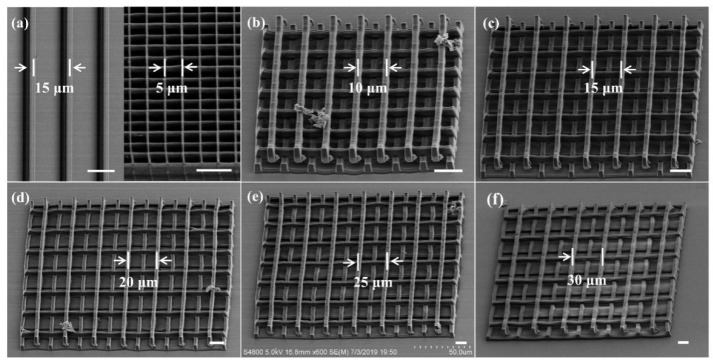
SEM images of different microstructures. (**a**) Hydrogel line array (left) and ST scaffold (right). (**b**–**f**) A series of the 3D microscaffolds with different strut spacings of 10 μm, 15 μm, 20 μm, 25 μm, and 30 μm, with corresponding porosities of 69.7%, 79.5%, 84.4%, 87.4% and 89.3%, respectively. The scale bar is 10 μm.

The geometry, dimension, strut parameters and pore size of scaffold play important roles in modulating cell growth, proliferation and migration, as previously reported [43]. To systematically investigate the relationship of the microstructures and cells, hydrogel micropattern line array, ST scaffold and 3D microscaffolds with pore size and strut spacing against cells were fabricated by TPP (Figure 4). The laser scanning speed was 150 μm/s and the average power (before the objective lens) was 5.6 mW. In the line array (left in Figure 4a), the line height, width and line spacing are 3.5 μm, 3 μm, and 15 μm, respectively. ST scaffold strut has a width of 0.5 μm, a pore size of 5 μm, and a total height of 9.6 μm. The partial enlarged view is shown in Figure S1. Figure 4b–f shows the SEM images of 3D microscaffolds with different porosities. The strut cross section is 3 μm × 3.5 μm (smaller than the cell seed). The distance between the struts was adjusted between 10 μm, 15 μm, 20 μm, 25 μm and 30 μm (larger than the cell seed but smaller than or equal to the spread cell), with corresponding porosities of 69.7%, 79.5%, 84.4%, 87.4%, and 89.3%. The individual scaffold is 200 μm × 200 μm in the transverse dimension and 14 μm in height. The refinement of the strut spacing against the cell in the 3D microscaffold enables us to study the influence of relative size design on regulation of cell morphology and behavior.

### 3.4. Modulation of Cell Behavior on Different Microstructures

**Figure 5:**
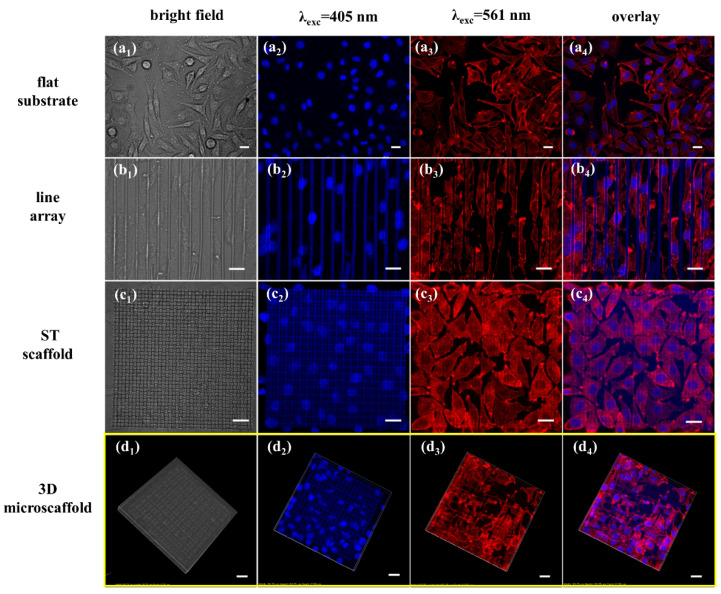
Bright field images, fluorescence images (excited by 405 nm and 561 nm) and overlay images of the cells on different microstructures. The overlay image was obtained from the fluorescence images of nuclei and F-actin. (**a_1_**–**a_4_**) Images on the flat substrate. (**b_1_**–**b_4_**) Images on the patterned substrate with line array. (**c_1_**–**c_4_**) Images on the ST scaffold with small pore size and thin strut. (**d_1_**–**d_4_**) Images on the 3D microscaffold with strut spacing of 20 μm. The scale bar is 20 μm.

Fibroblasts L929 were cultured on a flat substrate, a patterned substrate with line array, an ST scaffold and 3D microscaffolds to investigate how topographical cues affect cell behavior. Figure 5 shows the bright field images, fluorescence images and overlay images of the cells on different scaffolds. F-actin of L929 cell spreads in a random direction on the flat substrate in the shape of spindle-like, triangle-like and few star-like (Figure 5a_1_–a_4_). While on line patterned substrates, they were highly elongated and adaptively oriented along the lines (Figure 5b_1_–b_4_). Meanwhile, F-actin of L929 on the ST scaffold lost the slender tip of spindle-like and triangular-like shapes it possessed on the flat substrate, and it also did not have the obvious orientation as on the line array. Figure 5c_1_–c_4_ show less elongation of these cells. 

It is worth noting that the cells cultured on the 3D microscaffold show obvious differences in adhesion and distribution (Figure 5d_1_–d_4_). The cell nuclei that were embedded in the pore of the 3D microscaffold deformed under the influence of the strut showed an oblate spheroid shape. Cell nuclei adhered to the strut were in a semi-suspended state (the width of the strut is smaller than the cell seed). The images of F-actin demonstrated an orderly morphology that was strongly correlated with the scaffold. 

Substrate topology clues can significantly affect the shape of the cell, as well as attachment, migration, differentiation, etc. The L929 cells on the flat substrate appear in a strap-like shape, as Liu reported on a poly (vinyl alcohol)/poly (ethylene glycol) surface [44]. Cells cultured on the line array were oriented in alignment along the space formed between two struts, which was similar to the phenomena previously reported [45], appearing in a strip shape. When adhered to the ST scaffold with small pores and thin struts, the cells showed a less elongated and more spread-out microfilament cytoskeleton with many filopodia related to the strut (Figure S2). The patterns on these substrates are simple, and cannot simulate the complicated cell microenvironment in vivo. However, the 3D microscaffold showed stronger modulation of the cell. Thus, it is meaningful to explore the influence of parameters of the 3D microscaffold on cells.

### 3.5. Cell Behavior on a Series of 3D Microscaffolds

#### 3.5.1. The Effect of Porosity of 3D Microscaffolds on F-Actin

**Figure 6:**
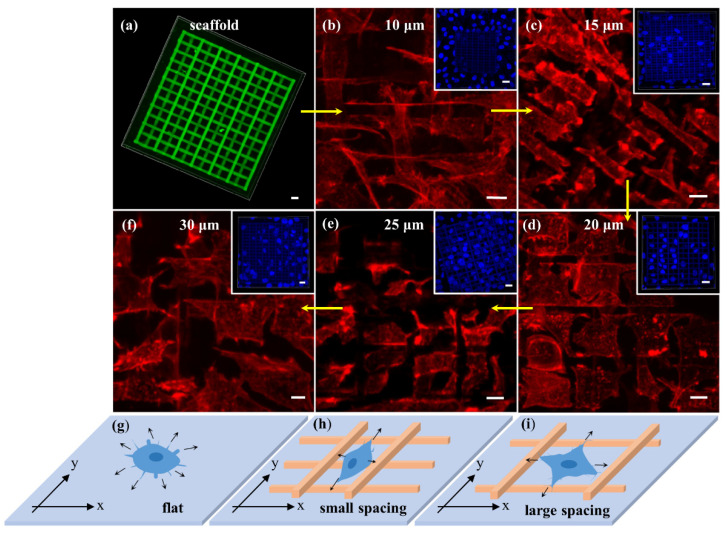
Confocal fluorescent microscopy images of (**a**) scaffold (excited by 488 nm) and cells grown on 3D microscaffolds with strut spacing of (**b**) 10 μm, (**c**) 15 μm, (**d**) 20 μm, (**e**) 25 μm, (**f**) 30 μm, respectively. (**g**–**i**) Schematic of the mechanism for regulating F-actin synthesis through sensing information by filopodia. Scale bar is 10 μm, while the scale bar in the inset is 20 μm.

Actin is important for inducing the transduction of signals that is essential for cell function and transmitting extracellular force [46]. Figure 6 illustrates F-actin of fibroblast L929 changes on a series of 3D microscaffolds with different porosity. Figure 6a shows the confocal fluorescence image of the 3D microscaffold, which is observed without staining with fluorescence probes. When the distance between the struts is as small as 10 μm (porosity of 69.7%), F-actin around the cell nucleus spreads in the space formed by the two adjacent struts, appearing as a long thin strip (Figure 6b). When the strut spacing is 15 μm (porosity of 79.5%), F-actin broadens along the strut axial and exhibits a rectangular shape (Figure 6c). As the strut spacing increases to 20 µm (porosity of 84.4%), the axial spreading of F-actin becomes more pronounced and starts to spread in a square shape (Figure 6d). Until the strut spacing increases to 25 µm (porosity of 87.4%) and 30 µm (porosity of 89.3%), F-actin shows a larger and more regular square shape (Figure 6e,f). F-actin changes regularly from a long strip to a square with changing porosity.

It’s well known that filopodia of cells can be used to perceive the surrounding physical and chemical environment, and transmit relevant information back to cells [47,48]. Once adhered to the surface, cells firstly extend filopodia around to perceive the environment, and the F-actin synthesis rate is the same in all directions before the filopodia touch any struts (Figure 6g). The 3D microscaffold has an intersecting architecture, and hence gives the cell different orientation possibilities. The 3D microscaffold is fabricated layer by layer, and for each layer, there are only parallel struts (Figure 6h,i). If the direction along the strut in the bottom layer is defined as the *x*-axis, the vertical direction is defined as the *y*-axis. When the strut spacing is small, for example, 10 μm, filopodia oriented in the *x*-axis first touch the front strut (Figure 6h) and regard it as an obstacle, then F-actin synthesis slows down or stops in the *x*-axis. Thus, there is less spread for F-actin in the *x*-axis. However, it spreads a long way, since there is no obstacle, in the *y*-axis. Thus, F-actin appears as a long strip shape. F-actin shows increased spread in the *x*-axis when the strut spacing is 15 μm. This is because the space in the *x*-axis increases compared to the spacing of 10 μm. Of course, F-actin in the *y*-axis decreases slightly owing to the limited capacity of material transfer in the cell within a certain period of time, making the F-actin a rectangular shape. Similarly, F-actin begins to change from rectangular to square (Figure 6i) when the strut spacing is 20 μm. This squareness is more pronounced when the strut spacing increases to 25 μm and 30 μm. This result further demonstrates that the change of F-actin is determined by the sensitivity and feedback of the filopodia. 

**Figure 7:**
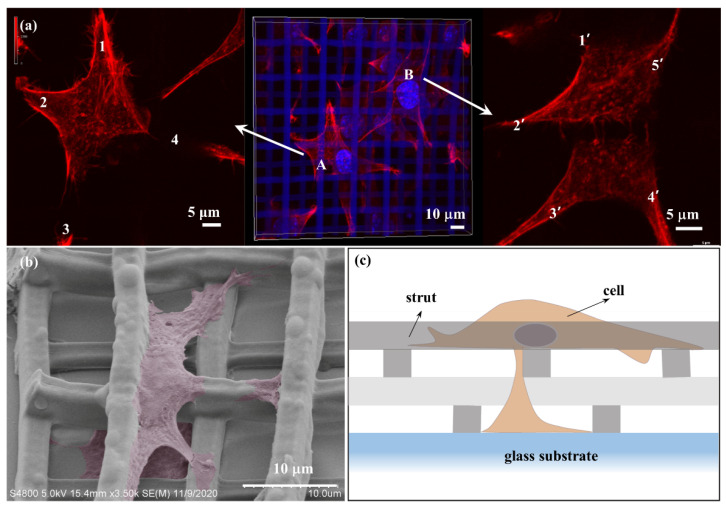
(**a**) “Starfish-like” F-actin with more lamellipodia on the scaffold with 20 μm strut spacing. (**b**) SEM image of lamellipodia extending from the cell body on 20 μm scaffold. (**c**) Schematic diagram of cell spreading in 3D microscaffold.

Lamellipodia play an important role in cell adhesion, migration and cell–cell interactions. Therefore, changes of lamellipodia in different scaffolds will affect the behavior of cells. Generally, fibroblasts L929 are in a spindle or triangular shape, with two or three lamellipodia. Cells on 3D microscaffolds have four, five, or even more lamellipodia (Figure 7a), and show a “starfish-like” microfilament cytoskeleton. Moreover, lamellipodia on 3D microscaffold can extend downward in the longitudinal direction along the struts, except spreading horizontally, thus exhibiting a perfect 3D shape (Figure 7b). Figure 7c is the schematic diagram of a cell in a 3D microscaffold. So far, few studies achieving 3D growth of single cells have been reported [49]. Different cell behaviors have been achieved by using 3D microscaffolds, which is very important for maintaining the biological functions of the cells in vivo. Furthermore, this would help to improve the efficiency of information exchange and material exchange between cells.

#### 3.5.2. The Influence of 3D Microscaffold on Cell Distribution

**Figure 8:**
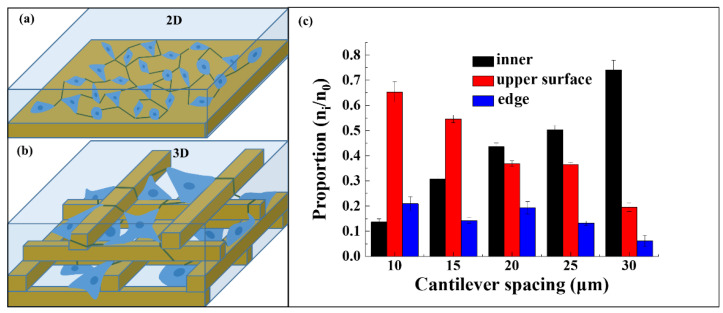
Schematic diagram of 2D (**a**) and 3D (**b**) communication networks. (**c**) Proportion of cells adhering to different positions, such as inner, upper surface, and edge of the 3D microscaffold.

The cells in various scaffolds often land in different ways and form a non-uniform cell distribution due to cell gravity, scaffold microporosity and surface chemistry, so static inoculation on the scaffold is still a challenge [50]. Cells on the micronano patterned surface belong to 2D growth. Filopodia and lamellipodia form a 2D attachment network along the substrate for material–information interaction and transmission (Figure 8a). In our 3D microscaffolds, cells automatically adhere to the different heights of the scaffold. This 3D static distribution can form a 3D interactive interaction network (Figure 8b). Such a transfer network would be much faster and more efficient than that of 2D network.

To evaluate 3D distribution of cells on 3D microscaffolds, the number of cells adhering to different locations was counted. Three main locations were included: the inner, the upper surface, and the edge of the 3D microscaffold, respectively. The chart of the 3D distribution of cells was analyzed using Origin Pro 9.1, and the data are expressed as mean and standard deviation derived from at least five sets of data. The proportion is the number of cells adhering to each location to the total number of cells on the whole scaffold. Cells falling into the scaffold increases with increasing strut spacing, from 13% (10 μm) to 77.8% (30 μm) (Figure 8c). On the contrary, cells on the upper surface of the scaffolds decrease from 62% (10 μm) to 17.8% (30 μm). Meanwhile, the cells adhering to the edge of the scaffold do not have regular changes because they are not related to the strut spacing. A strut spacing of 20 μm was found to be a critical point, and the proportion of cells falling into the inner and upper surface of this scaffold is close to 1:1. 

The pore size in 3D microscaffolds is half of the strut spacing. When the strut spacing is small, for example, 10 μm, the pore size is 5 μm, which is smaller than the cell seed (5–10 μm). Thus, the cell seeds scarcely fall inside the scaffold, but attach to the upper surface of the scaffold. The pore size is 7.5 μm when the strut spacing increases to 15 μm, which is larger than the cell seed. Therefore, 26% proportion of cell seeds with size smaller than 7.5 μm will fall inside the scaffold, while the vast majority of cells still adhere to the upper surface of the scaffold (58%). For the strut spacing of 20 μm, the pore size is 10 μm, which is similar to the size of the cell seed. In this case, it would no longer have a hindering effect on the cells, thus the ratio of the cells falling into the scaffold and on the upper surface is 1:1. When the scaffold strut spacing reaches 25 μm and 30 μm, the pore sizes are 12.5 μm and 15 μm, respectively. They are all larger than the cell seed, and thus cell seeds would fall into the pore and adhere to the bottom of the scaffold, but rarely adhere to the upper surface. Therefore, strut spacing of 20 μm in the 3D microscaffold is the critical size for 3D distribution of fibroblasts.

### 3.6. Cell–Cell and Cell–Scaffold Interactions on 3D Microscaffold

**Figure 9:**
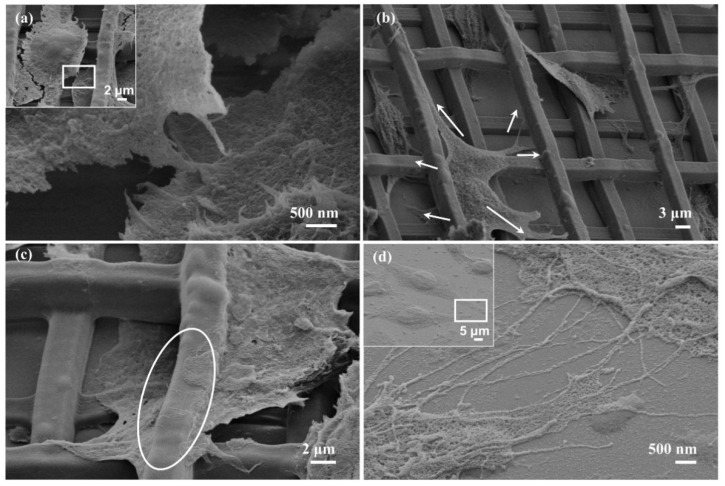
Cell–cell interactions in different forms. SEM images of the cell–cell interactions in (**a**) direct form, (**b**) long-distance remote form, and (**c**) strut-entangled form. (**d**) SEM images of the cell–cell interactions on the flat substrate.

There are three main ways that cells communicate information and transfer material within an organism: direct contact, channel transfer, and chemo secretion. In this study, cell–cell interactions in direct form, long-distance remote form and strut-entangled form were observed on the basis of SEM characterization (Figure 9). In the 3D microscaffold, cells adhering to different height positions can be observed to interact through direct contact (Figure 9a). Additionally, cells cross the height gradient to extend a majority of lamellipodia farther out for information exchange when the cell density within the scaffold is low and the distance between cells is large (Figure 9b). Figure 9c depicts the cells located on both sides of the strut extending their filopodia onto the strut. The filopodia connect together and entangle along the strut. When the cells adhere to flat substrate, the cells extend many filopodia, forming a 2D information–material channel network (Figure 9d). These results reveal that the cell interactions on 3D microscaffolds are much more complicated than those on 2D surfaces, which can help simulate the 3D cell microenvironment.

**Figure 10:**
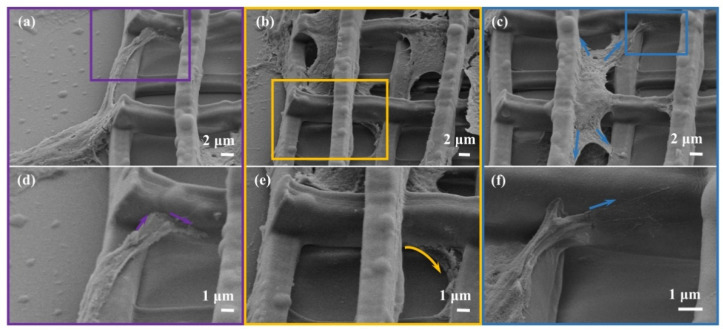
Cell–scaffold interactions. (**a**,**d**) SEM images of the cell on the flat surface climbing up to the edge of the 3D microscaffold. (**b**,**e**) SEM images of the cells inside the 3D microscaffold getting through the bottom of the strut. (**c**,**f**) SEM images of the cells inside the 3D microscaffold spreading up along the strut.

Since 3D scaffolds could provide a 3D environment for cells, it is important to understand the interactions between scaffolds and cells for tissue regeneration [51]. As shown in Figure 10, cells landing on the flat adjacent to the 3D scaffold climb up to the edge of the strut (Figure 10a). The front lamellipodium climbs over and extends along the upper surface when encountering the bottom strut. However, once encountering the upper vertical strut located directly in front, the lamellipodium fails to achieve the overturn, and grabs the side of the strut (Figure 10d). For cells on the edge of the scaffold (Figure 10b), the lamellipodium crosses both the height gradient and the lateral strut, like “legs” extending under the table (Figure 10e). Figure 10c illustrates the cells inside the scaffold extending the lamellipodium spreading up along the strut, also shown in Figure 10a. Interestingly, the front of the lamellipodium here is claw-shaped, and every “finger” is clear (Figure 10f). These results demonstrate that the 3D microscaffolds promoted the distribution of lamellipodia to three dimensions and thus induced population of cells towards a 3D distribution.

## 4. Conclusions

In summary, a series of PEGDA-based hydrogel microscaffolds were fabricated by two-photon polymerization. The dependence of the linewidth on laser power and scanning speed were thoroughly studied, and a best resolution of 87 nm was achieved. A Young’s modulus ranging from 65 to 92.1 kPa was obtained in water, revealing that the structure remained substantially stable in water phase. The noticeable relation between the behavior of fibroblast cell growth and the porosity in the architectures was demonstrated by LSCM and SEM. F-actin of cells on 3D microscaffold changed regularly from strip to square with the increase of strut spacing from 10 μm to 30 μm, while a turning point appears at 20 μm. More lamellipodia were observed for cells cultured on 3D microscaffolds, forming a 3D cell shape. Furthermore, this demonstrates that 3D distribution close to that found in in vivo conditions can be achieved by considering the relative size between cells and scaffold architecture. Overall, the fabricated 3D microscaffolds increase the surface area of the cells, which can be beneficial to the transfer of materials and information between cells. This study provides protocols for in vitro cell culture and cell biology, and thus would promote the development of tissue engineering and regenerative medicine.

## Data Availability

Not Applicable.

## Supplementary Materials

The following are available online at https://www.mdpi.com/article/10.3390/nano11092325/s1, Table S1: PEGDA-based hydrogel photoresist, Figure S2: SEM images of ST scaffold, Figure S3: Fibroblasts cultured on ST scaffold.
