# Supplementary material for: Modulation of Cell Behavior by 3D Biocompatible Hydrogel Microscaffolds with Precise Configuration
Source: Nanomaterials (Basel). 2021 Sep 7;11(9):2325. doi: 10.3390/nano11092325 (PMC8469000; doi:10.3390/nano11092325)
Supplement: Supplementary file 1 [file nanomaterials-11-02325-s001.zip › nanomaterials-1334037-supplementary-done.pdf]

nanomaterials

Supporting Information

Modulation of Cell Behavior by 3D Biocompatible Hydrogel Microscaffolds with Precise Configuration

Wei-Cai Zhang [1,2], Mei-Ling Zheng [1,2,*], Jie Liu [1], Feng Jin [1], Xian-Zi Dong [1], Min Guo [1,2] and Teng Li [1,2]

[1] Laboratory of Organic Nano Photonics and CAS Key Laboratory of Bio-Inspired Materials and Interfacial Science, Technical Institute of Physics and Chemistry, Chinese Academy of Sciences, No. 29, Zhongguancun East Road, Beijing 100190, China; zhangweicai17@mails.ucas.ac.cn (W.-C.Z.); liujieby@mail.ipc.ac.cn (J.L.); jinfeng@mail.ipc.ac.cn (F.J.); dongxianzi@mail.ipc.ac.cn (X.-Z.D.); hsdguom@163.com (M.G.); liteng19@mails.ucas.ac.cn (T.L.)

[2] School of Future Technologies, Yanqihu Campus, University of Chinese Academy of Sciences, Huaibei Zhuang, Huaibei Town, Huairou District, Beijing 101407, China

* Correspondence: zhengmeiling@mail.ipc.ac.cn; Tel.: +86-10-82543596

Contents

1. PEGDA-based hydrogel photoresist S-1

2. SEM images of STscaffold...S-2

3. Fibroblasts cultured on ST scaffold S-2

1. Formulation of PEGDA-based hydrogel photoresist

Table S1. Formulation of PEGDA-based (39.2 wt %) hydrogel photoresist.

Constituent	PEGDA	PE-3A	Photosensitizer	Photoinitiator
wt %	39.2	59.2	0.8	0.8

2-Benzyl-2-(dimethylamino)-1-[4-(4-morpholinyl)phenyl]-1-butanone was used as photosensitizer and Benzil was used as photoinitiator, which were 0.8 mg and 0.8 mg, respectively. PEGDA was used as monomer due to its good biocompatibility and PE-3A was used as crosslinking agent. PEGDA of 392 mg and PE-3A of 592 mg were used in our study.

2. SEM images of ST scaffold

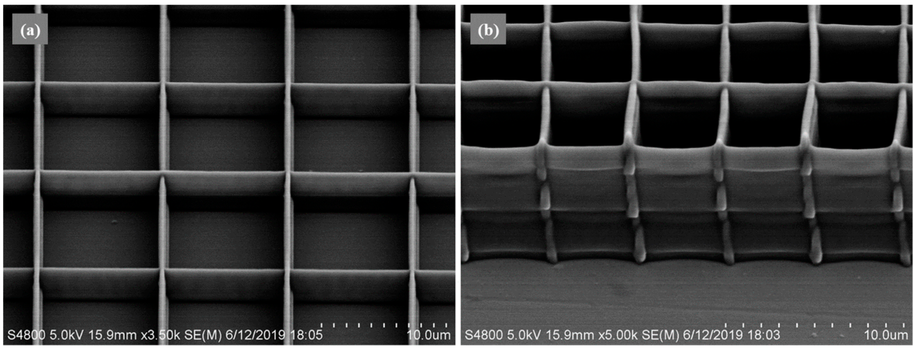

Figure S1. (a) Top view and (b) local details of SEM images for ST scaffold.

3. Fibroblasts cultured on ST scaffold

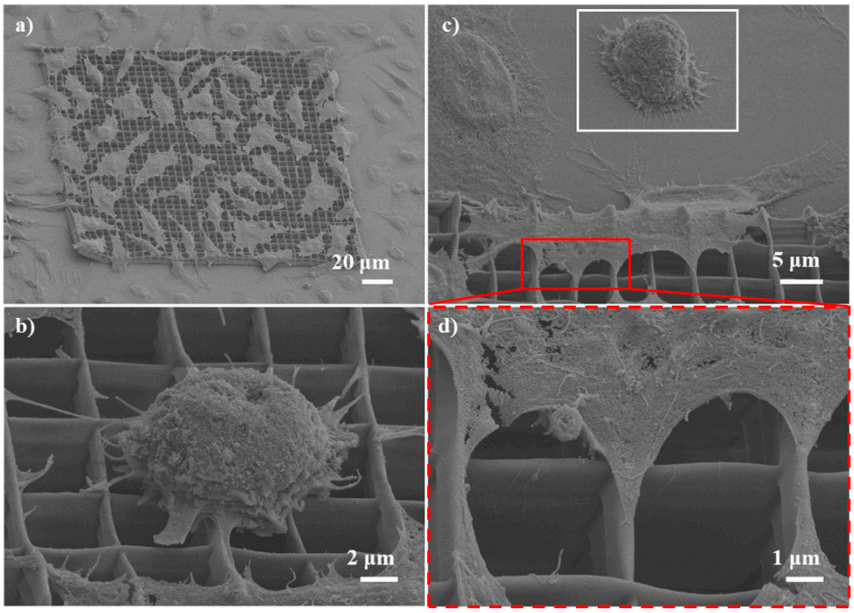

Figure S2. SEM images of fibroblasts cultured on ST scaffold for 48 hours. (a) Full view of cells on ST scaffold. (b) Cell on ST scaffold surface. (c) Spread cell on the edge of ST scaffold 2D flat substrate. (d) Magnification of spread cell in (c).

SEM image of the full view of cells on simple ST scaffold in Fig. S2a shows that there are more filopodia related to the cantilever at the edge of the cytoskeleton compared to those on flat substrate. For cell that fully adheres to the surface of ST scaffold, it stretches out a lot of long filopodia to perceive the environment before spreading (Fig. S2b). Fig. S2c describes that filopodia extend in all directions when the cell just adheres to the flat substrate and not spread. The difference is that the number of filopodia protruding from the cell on the ST scaffold is far less than that on flat substrate. Fig. S2d shows the filopodia extending along each cantilever in a similar triangular shape. Those confirm that small topographical parameter changes will significantly influence the cell behavior.
